# Subjective cognitive failures and their psychological correlates in a large Italian sample during quarantine/self-isolation for COVID-19

**DOI:** 10.1007/s10072-021-05268-1

**Published:** 2021-04-29

**Authors:** Gabriella Santangelo, Ivana Baldassarre, Andrea Barbaro, Nicola Davide Cavallo, Maria Cropano, Gianpaolo Maggi, Raffaele Nappo, Luigi Trojano, Simona Raimo

**Affiliations:** Department of Psychology, University of Campania “Luigi Vanvitelli”, 31 81100 Caserta, Viale Ellittico Italy

**Keywords:** COVID-19, Cognitive failures, Depression, Anger, Resilience, Quarantine

## Abstract

**Objective:**

The quarantine/self-isolation measures implemented to retard the spread of the 2019 coronavirus disease (COVID-19) may negatively affect the mental health of the population. The present study aimed to explore the impact of the psychological symptoms on the occurrence of cognitive failures in a large sample of home-dwelling Italian individuals during quarantine/self-isolation for COVID-19.

**Methods:**

We employed an online questionnaire using a virtual platform of Google Moduli. The questionnaire included an assessment of cognitive failures evaluated by the Perceived Memory and Attentional Failures Questionnaire (PerMAFaQ) and of resilience, coping style, depression, anger, and anxiety.

**Results:**

The online questionnaire was completed by 4175 participants revealing that about 30% of participants complained of cognitive failures at least sometimes during quarantine/self-isolation, whereas some respondents reported very frequent cognitive failures. Moreover, resilience was found to mediate the relationships between depressive and anger symptoms and cognitive failures. Although no difference was found on PerMAFaQ among smart-workers, non-smart-workers, and those currently not at work, people not working at the moment complained of more frequent cognitive failures.

**Conclusions:**

These findings indicate the need to implement psychological support intervention, particularly for vulnerable groups, to reduce anxiety, depression, and anger, and of psychoeducational interventions to enhance resilience reducing possible long-term cognitive consequences of the quarantine.

**Supplementary Information:**

The online version contains supplementary material available at 10.1007/s10072-021-05268-1.

## Introduction

In December 2019, the outbreak of a novel coronavirus disease (COVID-19) emerged in Wuhan of Hubei Province, China. Then, COVID-19 rapidly diffused to other countries becoming a public health emergency of international concern [1]. In Italy, in February 2020, the outbreak of COVID-19 started in the Lombardy region and spread throughout all regions. The Italian Government thus implemented extraordinary measures such as quarantine and social isolation at home, social distancing, and community containment from 10 March 2020.

Quarantine and isolation are unpleasant experiences leading to negative psychological effects including post-traumatic stress symptoms, confusion, and anger [2]. Studies focusing on the psychological distress of quarantined medical staff [1, 3, 4] reported exhaustion, anxiety, irritability, poor concentration and indecisiveness, reduced work performance, and reluctance to work until resignation. Other studies revealed that long periods of quarantine were associated with post-traumatic stress symptoms [5, 6], avoidance behaviors [1], and anger [1]. COVID-19 outbreak had led to psychological impact in Chinese [7–13], Spanish [14, 15], Turkish [16], and Italian [17–21] general population and medical staff [6, 7, 22] in the early stages of the COVID-19 epidemic. The evidence of the psychological impact of the COVID-19 on both medical staff and the general population suggests formulating psychological interventions to preserve the mental health of vulnerable groups during and after quarantine.

Taking into account that quarantine/isolation negatively impacts mental health [4], and prolonged distress can determine perceived deficits of memory and concentration [23], we designed a cross-sectional study to identify the impact of a long period (>1 month) of quarantine on cognitive status in a large Italian sample. We investigated psychological correlates of subjective cognitive failures, which are defined as subjective perceptions of lapses in cognition and reflect a pathological process occurring in the brain. In particular, we explored whether the occurrence of subjective cognitive failures was associated with anxiety, depression, and anger and whether the associations between cognitive failures and psychological reactions (i.e., anxiety, depression, and anger) were mediated by personal resilience (i.e., the capacity to thrive in the face of adversity, while maintaining relatively normal physical and psychological function over time) and/or by coping style (i.e., the employment of adaptive or maladaptive coping strategies to tolerate, minimize, accept, or ignore stressful situations). The identification of a possible mediating effect of personal resilience or/and coping style on the relationship between cognitive failures and psychological reactions could suggest the type of cognitive and psychological interventions most effective in preventing a cognitive decline after a long period of quarantine/self-isolation.

## Materials and methods

We adopted a cross-sectional survey methodology to assess psychological responses during the quarantine/self-isolation. We employed an online questionnaire using Google Moduli and disseminated it in virtual environments (i.e., Facebook, WhatsApp, and social virtual groups). In detail, since the Italian Government implemented extraordinary measures such as quarantine and social isolation at home, the online survey was disseminated to university students, friends, colleagues, and acquaintances; they were encouraged to pass it on to others. Therefore, we adopted a snowball sampling strategy to recruit a large Italian sample of people living in different Italian regions.

Participation in the survey was open from April 4 to April 26, 2020, i.e., during the period in which quarantine was issued by the Italian Government.

### Survey development

The structured questionnaire consisted of several sections: informed consent; sociodemographic data; subjective cognitive complaints; mental health status; personal resilience; and coping style.

To avoid missing data in data analysis, we applied the Google Moduli feature which allows you to make answers to questions mandatory. The only non-mandatory question was about the number of the rooms in the house.

#### Informed consent

The online survey started with a digital consent form describing the nature of the study. After individuals read the digital informed consent and agreed to participate, a subsequent web page was loaded.

#### Sociodemographic data

Gender, age, education, residential location, marital status, living status, household size, and employment status were collected. Respondents were asked to indicate if they had been admitted to the hospital in the previous month and had been tested for COVID-19. Contact history variables included close or indirect contact with an individual with confirmed COVID-19 and contact with an individual with suspected COVID-19. Respondents were asked to indicate the number of days spent in quarantine/self-isolation, housing characteristics, and previous psychiatric illnesses; respondents were also asked to rate the frequency of feeling boredom, frustration, and fear of getting infected with COVID-19.

#### Perceived Memory and Attentional Failures Questionnaire

Cognitive failures reflect a global liability towards frequent lapses in cognitive control. Several questionnaires have been developed to evaluate the severity or the frequency of these disturbances; however, some questionnaires include questions that evaluate cognitive failures for activities that could not be performed during quarantine (i.e., Cognitive Failures Questionnaire, CFQ) and others focus on memory failures only (i.e., Subjective Memory Questionnaire; Multifactorial Memory Questionnaire, MMQ; Memory Assessment Clinic-Q). Therefore, we developed a questionnaire including some items borrowed from available tools assessing memory changes (items 2, 7, 10 of Multifactorial Memory Questionnaire and item 6 of CFQ) plus specific items to assess perceived memory and attentional failures in everyday life activities performed at home (i.e., difficulty concentrating on the news of television or radio broadcasts; difficulty watching a movie until the end; difficulty concentrating while talking to someone else; difficulty focusing while reading a newspaper or a book; difficulty doing something at home because you end up doing something else without even realizing it). The questionnaire included 9 items. Each item was to be rated on a 5-point Likert scale ranging from 1 “never” to 5 “very often”. The total score ranged from 5 to 45 with higher scores indicating a higher propensity to cognitive failures.

#### Mental health status

Anxiety, anger, and depressive symptoms were assessed using the 7-item Generalized Anxiety Disorder Scale (GAD-7), the DSM-5 Level 2-Anger-Adult measure (DSM-5-Anger), and the Italian version of the Patient Health Questionnaire-9 (PHQ-9). For the reference and description of the tools, see Supplemental Material 1.

#### Resilience and coping style

Individual response to stressful situations and coping style were assessed using the Italian translation of the Brief Resilience Scale (BRS) and the Coping Scale, respectively. The several steps of linguistic validation of the scales are described in Supplemental Material 1.

### Statistical analysis

#### Psychometric properties of the PerMAFaQ, BRS, and Coping Scale

Comprehensibility, internal consistency and construct validity of the scales were evaluated on 109 individuals (mean age: 43.1, SD=10.5, range 26-80; mean education level: 15, SD=2.9, range = 8-18; male=29; females=80) recruited through Facebook and WhatsApp and not included in the final sample. The individuals completed the online questionnaires and an online comprehension test, where each participant had to answer affirmatively (=1) if he/she evaluated the item and the answers as easy to understand, or negatively (=0) if he/she evaluated the item and the responses as difficult to understand. A comprehension rate was obtained as the percentage of questions and pre-coded answers of all items correctly understood by all participants [24]. We evaluated internal consistency by Cronbach’s alpha (value of 0.6-0.7 indicates an acceptable level of reliability) and the structure of the scales by principal component analysis (PCA) with VARIMAX rotation; to identify the number of independent components, we applied the eigenvalue >1 criterion since it seems to be less subjective and arbitrary to interpret than the scree plot criterion.

#### Relationship between cognitive failures and sociodemographic aspects, mental health status, resilience, and coping style

Descriptive statistics were calculated for sociodemographic characteristics and all psychological variables. We carried out simple and multiple linear regressions to evaluate the association of the subjective cognitive failures with the following: sociodemographic characteristics such as age, sex (coded as male=1; female=0), educational level (coded as elementary school=0, middle school=1, high school=2, degree and post-degree=3), duration of the quarantine/self-isolation, number of children in the house, number of rooms in a house, number of individuals per house, the ratio between the number of individuals and number of rooms in a house, number of exits from home in a week, indirect contact with people affected by COVID-19, fear of getting infected with COVID-19, coping style and personal resilience, depression, anxiety, and anger.

To investigate if and how resilience and coping strategies mediated the relationship between the mental health status and subjective cognitive failures, we carried out a mediation analysis entering mental health status variables significantly associated with cognitive failures in multiple regression analysis as independent variables, cognitive failures as a dependent, and resilience and coping style scale as mediators.

To evaluate the significance of direct, indirect, and total effects, bootstrapping procedure with 5000 samples with replacement from the full sample to construct bias-corrected 95% confidence intervals was conducted by SPSS Macro PROCESS.

To evaluate whether subjective cognitive failures are associated with occupational attainment (i.e., unemployed versus smart-workers versus workers at the office), and healthcare work (i.e., physicians, nurses, and others versus non-healthcare workers), we performed multivariate analysis of variance (MANOVA), with Bonferroni post hoc tests. The significance level was set at *α* = .05.

Statistical analysis was performed using SPSS Statistic 21.0.

## Results

### Comprehensibility and the psychometric properties of the PerMAFaQ, BRS, and Coping Scale

The items of the Perceived Memory and Attentional Failures Questionnaire (PerMAFaQ) were rated as easy to understand and the response modality as easy to comply by 97% and 98% of the testing participants, respectively. The items of the Italian version of BRS were rated as easy to understand and the response modality as easy to comply by 96% and 98% of the testing participants, respectively. The items of the Italian version of the Coping Scale were rated as easy to understand and the response modality as easy to comply by 95% and 98% of the testing participants, respectively. Cronbach’s alpha was higher than 0.7 for all scales (Supplemental Material 2.A). The mean scores of PerMAFaQ, BRS, and Coping scales were 17.1 (SD=6.5), 21.1 (SD=4.7), and 33.4 (SD=5.1) respectively, showing high internal consistency, as assessed by Cronbach’s alpha, for PerMAFaQ (0.883), BRS (0.878), and Coping Scale (0.738).

The PCA for PerMAFaQ revealed 2 eigenvalues exceeding 1, accounting for 64.8% of variance. Scree plot revealed a two-factor model, which provided the best fit; the first factor (F1) explained 52.3% of variance and included items related to attentive failures (items: 2, 3, 4, 5, 6). The second factor (F2) explained 12.5% of variance and included items representing memory failures (items: 1, 7, 8, 9) (Supplemental Material 2.B).

The PCA for BRS revealed 1 eigenvalue exceeding 1, accounting for 62.8% of variance, indicating the unifactorial structure of the scale.

The PCA for Coping Scale revealed 4 eigenvalues exceeding 1, accounting for 61.6% of variance. The first factor (F1) explained 26.3% of variance and included items related to the capacity of seeing the positive side of the situation, seeing the humor in it (items: 2, 5, 8, 10). The second factor (F2; related to the capacity of thinking about the problem from a different point of view, spending time trying to understand what happened), the third factor (F3, related to changing habits and lifestyle), and the fourth factor (F4; related to the capacity of making compromises and waiting problem out) explained 13.8%, 12.2%, and 9.3% of variance (Supplemental Material 2.C).

### Survey respondents

The survey was completed by 4175 subjects (Table 1). There was no missing data in our data analysis. Most participants were women (70.1%), aged 18-30 years (44.3%), had an educational level of graduation (57.2%) and were employed (63.6%).

**Table 1:** Sociodemographic data of the sample

Variables					
Age	18-30	31-40	41-50	51 or more	
	1850 (44.3%)	912 (21.8%)	587 (14.1%)	826 (19.8%)	
Sex	Female	Male			
	2928 (70.1%)	1247 (29.9%)			
Level of education	Elementary	Middle school	High school	Degree and post-degree	
	12 (0.3%)	179 (4.3%)	1597 (38.2%)	2387 (57.2%)	
Marital status	Married	Unmarried/maiden	Divorced/separated	Widower	
	1370 (32.8%)	2520 (60.4%)	241 (5.8%)	44 (1%)	
Number of people per household	Alone/1	2	3-5	6 or more	
	515 (12.3%)	871 (20.9%)	2649 (63.4%)	140 (3.4%)	
Number of children per household	0	1-2	3-5	6 or more	
	3145 (75.3%)	884 (21.2%)	119 (2.9%)	27 (0.6%)	
Number of rooms in a house	1-2	3	4	5 or more	
	376 (9%)	789 (18.9%)	1200 (28.7%)	1810 (43.4%)	
House with…	1 or more windows	Outdoor space (terrace, balcony, garden, or shared courtyard)			
	250 (6%)	3925 (94%)			
Employment status	Unemployed	Students	Employed	Retired	
	382 (9.1%)	1014 (24.3%)	2654 (63.6%)	125 (3%)	
Healthcare workers	Physicians	Nurses	Other (e.g., psychologists, laboratory technicians, or medical waste handlers)	Non-medical workers	
	119 (2.9%)	71 (1.7%)	380 (9.1%)	3605 (86.3%)	
Duration of quarantine/self-isolation	Mean (SD)	Median			
	31.37 (5.8)	30			
Work modalities	Smart-working	Office	No job		
	1285 (30.8%)	513 (12.3)	2377 (56.9%)		
Number of go out in the last week	0	1-2	3-4	5 or more	
	1294 (31%)	2136 (51.2%)	355 (8.5%)	390 (9.3%)	
Being affected by COVID-19	No	Yes (1 symptomatic; 3 asymptomatic)	I do not answer	No (symptomatic but not tested by swab)	Yes (remitted)
	4052 (97%)	4 (0.1%)	59 (1.4%)	49 (1.2%)	11 (0.3%)
Direct contact with people affected by COVID-19	I do not answer	No	Yes		
	63 (1.5%)	3929 (94.1%)	183 (4.4%)		
Indirect contact with people affected by COVID-19	I do not answer	No	Yes		
	30 (0.7%)	2406 (57.6%)	1739 (41.7%)		
Diagnosis of psychopathology	I do not answer	No	Yes		
	62 (1.5%)	3899 (93.4%)	214 (5.1%)		
Boredom	Never	Sometimes	Often	Always	Mean (SD)
	906 (21.7%)	1430 (34.2%)	1135 (27.2%)	704 (16.9%)	2.39 (1.00)
Frustration	Never	Sometimes	Often	Always	Mean (SD)
	1081 (25.9%)	1168 (28%)	1095 (26.2%)	831 (19.9%)	2.4 (1.07)
Fear of getting infected with COVID-19.	Never	Sometimes	Often	Always	Mean (SD)
	908 (21.8%)	1520 (36.4%)	1016 (24.3%)	731 (17.5%)	2.38 (1.01)
Scales	Mean	SD			
BRS score	20.2	4.3			
Coping Scale	34.4	5.1			
GAD-7	13.8	4.1			
DSM-5-Anger	49.9	10.1			
PHQ-9	16.1	4.5			

Frequency (percentage)

*SD* standard deviation; *BRS* Brief Resilience Scale; *GAD-7* 7-item Generalized Anxiety Disorder Scale; *PHQ-9* Patient Health Questionnaire-9; *DSM-5-Anger* DSM-5 Level 2-Anger-Adult measure

During the quarantine/self-isolation (mean duration was 31.37 days), a fair percentage of participants experienced boredom (*n* = 3269, 78.3%), frustration (*n* = 3094, 74.1%), and fear of getting infected with COVID-19 (*n* = 3267, 78.2%).

Clinically significant GAD evaluated by GAD-7 occurred in 861 participants (20.6%). Mild, moderate, and severe levels of anxiety on the GAD-7 occurred in 2121 (51%), 621 (15%), and 240 (6%), whereas 1202 participants (29%) had no anxiety symptoms. Anger evaluated by DSM-5-Anger was absent or occasional in 2626 (63%) respondents, mild in 891 (21%), moderate in 574 (14%), and severe in 84 (2%). Out of the respondents, 16% experienced clinically significant anger.

Depressive symptomatology evaluated by PHQ-9 was absent or occasional in 1260 (30%) respondents, subthreshold in 1853 (44%), mild in 783 (19%), moderate in 212 (5%), and severe in 67 (2%) subjects.

The depressive symptom “Have you ever had a fit of tears” was rated as never occurring by 2531 (60.6%) respondents, as occurring some days by 1487 (35.6%) respondents, as occurring in most of the time by 93 (2.2%) respondents, and as occurring “nearly every day” by 64 (1.5%) respondents.

### Perceived memory and attentional failures

An average of 27.5% of the participants complained of cognitive failures during quarantine/self-isolation (Table 2). The mean score on PerMAFaQ was 17.40 (SD: 6.21; median: 16; 25th percentile = 12, 75th percentile = 21, 95th percentile = 29). Dividing the whole sample according to the median, 2141 had a total score from 0 to 16, whereas 2034 had a total score above 16. Item 8 had the highest mean score (2.20), whereas item 2 had the lowest mean score (1.66).

**Table 2:** Descriptive of items included in the Perceived Memory and Attentional Failures Questionnaire

Items	Never	Rarely	Sometimes	Often	Always	Sometimes to Always
In the period of the quarantine/self-isolation…						
1. Do you have trouble remembering where you left your things (e.g., glasses, keys, mobile phone)? (item 2 of MMQ)	1657 (39.7%)	1309 (31.3%)	910 (21.8%)	225 (5.4%)	74 (1.8%)	29%
2. Do you have trouble remembering the contents of newspapers, newscasts, and newsletter? (item 10 of MMQ)	2227 (53.3%)	1241 (29.7%)	651 (15.6%)	3 (0.1%)	53 (1.3%)	17%
3. Do you have trouble focusing on the news you hear on television or radio broadcasts?	2215 (53.1%)	1104 (26.4%)	682 (16.3%)	123 (3%)	51 (1.2%)	20.5%
4. Do you have trouble watching a movie from start to the end?	2109 (50.5%)	1045 (25%)	675 (16.2%)	255 (6.1%)	91 (2.2%)	24.5%
5. Do you have trouble focusing while talking to someone?	2088 (50%)	1224 (29.3%)	710 (17%)	118 (2.8%)	35 (0.8%)	20.6%
6. Do you have trouble focusing while reading a newspaper or a book?	1533 (36.7%)	1262 (30.2%)	938 (22.5%)	330 (7.9%)	112 (2.7%)	33.1%
7. Do you have trouble doing something at home because you end up doing something else without even realizing it?	1653 (39.6%)	1161 (27.8%)	902 (21.6%)	341 (8.2%)	118 (2.8%)	32.6%
8. Has it happened to you to forget the reason why you went from one part of the house to another? (item 7 of MMQ)	1224 (29.3%)	1380 (33.1%)	1196 (28.6%)	268 (6.4%)	107 (2.6%)	37.6%
9. Has it accidentally happened to you to leave the light or the television on in a room? (item 6 of CFQ)	1558 (37.3%)	1255 (30.1%)	1000 (24%)	244 (5.8%)	118 (2.8%)	32.6%
						Mean 27.5%

*CFQ* Cognitive Failures Questionnaire; *MMQ* Multifactorial Memory Questionnaire

Simple linear regression analyses revealed the following: a significant and negative relationship between PerMAFaQ score and sex (*p*<0.001), level of education (*p*<0.001), number of people in the house (*p* = 0.002), number of rooms (*p* = 0.005), BRS (*p*<0.001), and Coping Scale (*p*<0.001); a significant and positive relationship between PerMAFaQ score and age (*p* = 0.052), duration of quarantine/self-isolation (*p* = 0.040), number of exits from home in a week (*p* = 0.019), fear of getting infected with COVID-19 (*p*<0.001), and score on PHQ-9 (*p*<0.001), GAD (*p*<0.001), and DSM-5-Anger (*p*<0.001) (Table 3). A multiple regression analysis (where age, sex, level of education, number of people in the house, duration of quarantine/self-isolation, number of exits from home in a week, number of rooms, fear of getting infected with COVID-19, PHQ-9, GAD, and DSM-5-Anger were entered in block 1 and scores on BRS and coping style scale were entered in block 2) revealed that a higher score on PerMAFaQ was significantly related to more advanced age (*p*<0.001), lower educational level (*p*<0.001), female sex (*p* = 0.001), more people in the house (*p*<0.001), more exits per week (*p*<0.001), lower scores on BRS (*p*<0.001), and higher scores on PHQ-9 (*p*<0.001), and DSM-5-Anger (*p*<0.001) (Table 3).

**Table 3:** Results from simple and multiple regression analyses: Perceived Memory and Attentional Failures Questionnaire score is computed as dependent variable

	Simple				Multiple			
			95% confidence limits				95% confidence limits	
	Beta	*t*	Lower	Upper	Beta	*t*	Lower	Upper
Age	.03	1.94	.001	.03	.17	12.37***	.06	.09
Sex	−.13	−8.34***	−2.15	−1.33	−.04	−3.31**	−.92	−.24
Level of education	−.08	−5.07***	−1.14	−.50	−.05	−3.70***	−.74	−.23
Duration of self-isolation	.03	2.06*	.002	.08	-	-	-	-
Number of people in a house	−.05	−3.04**	−.64	−.14	−.06	−4.41***	−.68	−.26
Number of children in a house	−.001	−0.04	−.35	.34				
Number of rooms in a house	−.04	−2.79**	−.46	−.08	-	-	-	-
Ratio between people and rooms in a house	.003	.20	−.48	.59				
Number of outgoings a week	.04	2.34*	.04	.47	.05	3.97***	.19	.55
BRS	−.36	−25.25***	−.56	−.48	−.12	−8.61***	−.21	−.13
Coping style scale	−.11	−7.02***	−.17	−.10	-	-	-	-
Infected people	.03	1.65	−.01	.11				
Fear of getting COVID-19	.14	8.85***	.65	1.02	-	-	-	-
PHQ-9	.56	43.18***	.74	.81	.45	27.21	.58	.67
GAD-7	.45	32.13***	.65	.73	-	-	-	-
DSM-5-Anger	.43	30.33***	.25	.28	.14	8.66	.07	.11

*BRS* Brief Resilience Scale; *PHQ-9* Patient Health Questionnaire-9; *GAD-7* 7-item Generalized Anxiety Disorder Scale; *DSM-5-Anger* DSM-5 Level 2-Anger-Adult

p<0.05*, p<0.01**, p<0.001***

#### Mediation analysis

Based on the abovementioned multiple regression analysis, we designed a mediation model to test the mediator effect of resilience (BRS) on the relationship between depression (PHQ-9) and cognitive failures (PerMAFaQ) and the relationship between anger symptoms (DSM-5-Anger) and cognitive failures (PerMAFaQ). More severe symptoms of depression and anger were related to poorer resilience (depression: *B* = −0.328; *p*<0.001; anger: *B* = −0.082; *p*<0.001). Subsequently, poorer resilience was related to more cognitive failures (*B* = −0.178; *p*<0.001).

**Fig. 1:**
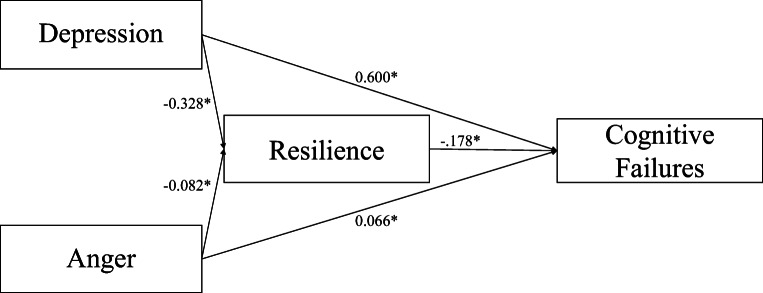
Scheme of the mediation effects of resilience in the relationship between mental health status and cognitive failures. *p < .05

The 95% bias-corrected CI based on 5000 bootstrap samples revealed significant direct (depression: estimate effect: 0.600; 95% CI: 0.614–0.703; anger: estimate effect: 0.066; 95% CI: 0.046–0.086) and total effects (depression: estimate effect: 0.658; 95% CI: 0.614–0.703; anger: estimate effect: 0.081; 95% CI: 0.061–0.101) of depression and anger symptoms on cognitive failures. However, the indirect effect of depressive symptoms on cognitive failures through resilience abilities (estimate effect: 0.058; 95% CI: 0.614–0.703) and the indirect effect of anger on cognitive failures through resilience (estimate effect: 0.015; 95% CI: 0.010–0.019) were both significant indicating a mediator effect of resilience for both the relationship between depressive symptoms and cognitive failures and the relationship between anger and cognitive failures (Fig. 1).

Furthermore, to further test the robustness of this model, we performed a mediation analysis entering coping strategies as the mediator of the associations of depression (PHQ-9) and anger (DSM-5-Anger) with cognitive failures (PerMAFaQ).

We found that more severe symptoms of depression and anger were related to poorer coping strategies (depression: *B*=−0.124; *p*<0.001; anger: *B*=−0.060; *p*<0.001), whereas no significant association was found between coping strategies and cognitive failures (*B*=0.006; *p*=0.734).

The 95% bias-corrected CI based on 5000 bootstrap samples revealed significant direct effects of depression (estimate effect: 0.659; 95% CI: 0.615–0.703) and anger (estimate effect: 0.081; 95% CI: 0.061–0.101) on cognitive failures, whereas the indirect ones through coping strategies were not (depression: estimate effect: −0.001; 95% CI: −0.005 to 0.004; anger: estimate effect: −0.000; 95% CI: −0.002 to 0.002) indicating no mediator effect of coping strategies for the relationships between depressive symptoms and cognitive failures and the relationship between anger and cognitive failures.

### Comparisons between groups

#### Cognitive failures in healthcare workers

We enrolled 570 healthcare workers (i.e., physicians, nurses, and other types such as psychologists, laboratory technicians, or medical waste handlers) and 3605 non-health workers. The two groups did not differ on PerMAFaQ (Table 4). We found an average percentage of 25.9% of the healthcare workers and 27.7% of the non-healthcare workers who complained of cognitive failures (chi-square = 0.94, *p* = 0.33; Supplemental Material 3.A).

**Table 4:** Comparisons on Perceived Memory and Attentional Failures Questionnaire (PerMAFaQ) between healthcare workers and non-healthcare workers and among people who work at the office, people who work by smart-working, and people who do not work during quarantine/self-isolation

	PMAFQ (mean ± SD)	*F*	*p*	*η*^2^*p*
Healthcare workers	16.93 ± 6.11	3.74	.05	.001
Non-healthcare workers	17.47 ± 6.22			
People who work at office	17.27 ± 6.28	1.57	.21	.001
People who work by smart-working	17.18 ± 6.41			
People who do not work	17.54 ± 6.08			

#### Cognitive failures among smart-workers, non-smart-workers, and those currently not at work

We identified 1285 smart-workers, 513 non-smart-workers, and 2377 respondents who were not currently at work. The three groups did not differ on PerMAFaQ (Table 4), but complaints of cognitive failures were significantly more frequent in people not working at the moment (28.2%) than in smart-workers or non-smart-workers (26.5%; Supplemental Material 3.B).

## Discussion

The present study revealed that about 30% of participants complained of cognitive failures (i.e., attentive and memory difficulties) at least sometimes during quarantine/self-isolation; some respondents referred to very frequent cognitive failures. The prevalence rate of cognitive failures in our study is higher than that reported in previous population-based studies on people aged 18-92 years, in which values of 22% [25] and 10.7% have been reported [26]. Although we had no control sample, the high prevalence of subjective cognitive failures in the present study compared to previous evidence would suggest that the long period of quarantine/self-isolation was at least indirectly correlated with the feeling of reduced cognitive efficiency.

The regression analyses provided some insights into the psychological mechanisms associated with self-perceived cognitive failures. Indeed, multiple regression analysis revealed an independent association of cognitive failures with female gender, and advancing age consistently with the literature on the relationship between age and cognitive decline [27]. More frequent cognitive failures were associated with lower educational level, fewer people in the house, and more exits a week. As high educational level and more active social lifestyle are proxies of cognitive reserve related to cognitively and socially stimulating lifestyles [28], which contrasts cognitive changes related to the aging process, our results might be compatible with the idea that people with low levels of cognitive reserve are more liable to develop self-perceived cognitive failures than people with high levels of cognitive reserve. A higher frequency of cognitive failures was independently associated with lower levels of resilience and more severe depressive symptoms and anger.

Faced with this complex picture, we investigated the possible role of personal resilience and coping style as mediators of the relationship between perceived cognitive failures and psychological symptoms using mediation analyses. We could observe that the relationships of depressive symptoms and anger on cognitive failures were mediated by resilience abilities rather than coping strategies. Indeed, the protective role of resilience during the COVID-19 has been widely reported [29–31], whereas it is known that the effectiveness of adopting coping strategies is not the same in different types of stressful situations since it depends on the type of stressor [32–34]. In summary, resilience could represent a protective factor reducing the impact of depression and anger on the development of subjective cognitive failures, which were quite frequent in people with low cognitive reserve.

Considering that subjective cognitive complaints could last even after quarantine and are a risk factor of developing a faster cognitive decline [35], our results suggested the need for psychoeducational interventions to promote and encourage people to foster resilience and to practice mental and physical exercise and to maintain frequent social relationships, possibly using new technologies during periods of isolation. This issue is relevant to help people to increase cognitive reserve and contrast cognitive changes related to aging and an acute stressful situation. Cognitive changes due to acute stress may be interpreted in light of a great sensitivity of the hippocampus to stress that is revealed by the profound suppression of hippocampal synaptic plasticity after acute exposure to stressors [36–40] or increased glucocorticoids [40]. In addition to the hippocampus, evidence indicates that stress-induced memory impairments can also be a consequence of alterations of dopaminergic or noradrenergic [41, 42] transmissions in brain structures such as the prefrontal cortex involved in high-order cognitive functions (e.g., working memory and executive function).

Whereas previous studies explored psychological reactions in healthcare workers [22, 43], we compared healthcare and non-health workers on cognitive symptoms, without finding any significant difference between the two groups. These findings could support the idea that quarantine had an impact on cognitive functioning independently from the type of occupational attainment. Consistent with these data, we observed that smart-workers and non-smart-workers did not show any difference on the cognitive failures questionnaire. Only people who were not working at the moment showed higher frequencies of cognitive failures. During the lockdown period, work on some activities was carried out through smart-working, whereas for other activities, the closure led some people to stop working. The change was sudden and it could have led to serious cognitive reactions, but we did not detect differences related to the working status. As this study was closed at the end of the so-called phase 1 of COVID-19 emergency, it is highly plausible that more substantial differences related to the working status and its connected socio-economic variables will eventually emerge in the following weeks.

Beyond targeting perceived cognitive failures, our study provided an overview of psychological distress during long-lasting quarantine/self-isolation. Feelings such as boredom, frustration, and fear of getting infected occurred in more than 70% of the Italian sample. These feelings have not been investigated before altogether. Fear of contagion has been explored in a study performed in South Korea by online survey 4 weeks after confirmation of the first case of COVID-19 [44]. In that study, 51.3% of the respondents believed that their perceived chance of infection (perceived susceptibility) were relatively low, whereas our study revealed that about 40% of the respondents perceived always or often the fear of getting infected with COVID-19. This observation might suggest that quite large variability exists across countries and cultures in the perceived risk of infection during pandemics, and this might be highly relevant to comprehend people’s adhesion to government lockdown measures. Such variability would not seem to be explained by differences in variables assessing mental health status.

The present study is characterized by several limitations. Firstly, it is a cross-sectional study on the associations between quarantine/self-isolation and subjective cognitive functioning; we have no data regarding cognitive functioning before the outbreak of COVID-19 or after easing of lockdown in the same sample or other kinds of controls. The respondents were recruited by a snowball strategy not balanced on a priori basis, and this could limit generalization of the results, notwithstanding the large size of the sample. We had to employ a novel specific questionnaire for assessing subjective cognitive failures, but the PerMAFaQ was tailored to evaluate cognitive failures occurring at home specifically, as people spent more time at home for the outbreak of COVID-19. We got evidence that the questionnaire was simple to be administered and comprehended and had acceptable internal consistency, as observed for the Italian translations of the BRS and Copying Scale. Nonetheless, the psychometric properties of these tools should be further investigated.

The present study suggested the need to implement psychological support intervention, particularly for vulnerable groups [14] to reduce psychological symptoms, and of psychoeducational interventions to foster resilience and reduce possible long-term cognitive consequences of the quarantine.

## Supplementary information

ESM 1 (DOCX 16 kb).

ESM 2 (DOCX 17 kb).

ESM 3 (DOCX 16 kb).

## Data Availability

Data and materials will be shared on request.
