# Supplementary material for: Subjective cognitive failures and their psychological correlates in a large Italian sample during quarantine/self-isolation for COVID-19
Source: Neurol Sci. 2021 Apr 29;42(7):2625–35. doi: 10.1007/s10072-021-05268-1 (PMC8082482; doi:10.1007/s10072-021-05268-1)
Supplement: Supplementary file 1 — (DOCX 16 kb). [file 10072_2021_5268_MOESM1_ESM.docx]

**Supplemental material 1. References and description of tools**

*Mental health status*

Anxiety symptoms were assessed using the 7-item Generalized Anxiety Disorder scale (GAD-7; [1]) assessing the DSM-IV symptoms for Generalized Anxiety Disorder [2]. Each item was rated on a 4-point Likert scale ranging from 0 ‘never’ to 3 ‘nearly every day’. Total score ranged 0-21; a score equal to or greater than 10 identifies GAD, whereas cut-off points of 5, 10, and 15 indicated mild, moderate, and severe levels of anxiety.

1. Spitzer RL, Kroenke K, Williams JB, et al. A brief measure for assessing generalized anxiety disorder: the GAD-7. Arch Intern Med. 2006; 22;166(10):1092-7. doi: 10.1001/archinte.166.10.1092
2. American Psychiatric Association. Diagnostic and Statistical Manual of Mental Disorders (4th ed.).1994. Washington, DC: American Psychiatric Association.

Anger was assessed using the DSM-5 Level 2-Anger-Adult measure, a 5-item version of the PROMIS Anger Short Form assessing severity of an individual’s anger during the past 7 days (DSM-5-Anger). Each item was rated on a 5-point Likert scale ranging from 1 ‘never’ to 5 ‘always’. Total score ranged from 5 to 25; a higher total score indicates greater anger severity. The total score has to be converted in T-score which is interpreted as follows: Less than 55= None to slight; 55.0-59.9= Mild; 60.0-69.9= Moderate; 70 and over= Severe anger. For the Italian version of the scale, the code is Code IT/CPSZ/03 DSM5 Rabbia Livello 2 Adulti and is available in <https://www.psicologozatelli.it/dsm5-test-in-italiano/>

1. American Psychiatric Association. Diagnostic and statistical manual of mental disorders (5th ed.). 2013. Washington, DC: American Psychiatric Association.

Depressive symptoms were evaluated using the Italian version of the Patient Health Questionnaire-9 (PHQ-9; [4,5]), a self-report 9-item inventory exploring symptoms of a major depressive episode as defined by the DSM-5. Each item was rated on a 4-point Likert scale ranging from 0 ‘not at all’ to 3 ‘nearly every day’. The total score ranged from 0 to 27; a cut-off score ≥ 10 indicated a clinically significant depression.

We used the translated versions of the GAD-7 and PHQ-9 available on the following website: <https://www.phqscreeners.com/select-screener>, which were shown to be valid and invariant across different sex, patient strata, and languages.

Last, we added one statement “Have you ever had a fit of tears” to assess further depressive symptomatology. This item was rated on a 4-point Likert scale ranging from 0 ‘not at all’ to 3 ‘nearly every day’.

1. Kroenke K, Spitzer RL, Williams JB. The PHQ-9: validity of a brief depression severity measure. J Gen Intern Med. 2001;16(9):606-13. Doi: 10.1046/j.1525-1497.2001.016009606.x
2. Mazzotti E, Fassone G, Picardi A, et al. The Patient Health Questionnaire (PHQ) for the screening of psychiatric disorders: a validation study versus the Structured Clinical Interview for DSM-IV axis I (SCID-I). Ital J Psychopathol 2003;9: 235-42.

Resilience

Individual response to stressful situations was assessed by means of the Brief Resilience Scale (BRS, [6]), including 6 items. Each item was rated on a 5-point Likert scale ranging from 1 ‘strongly disagree’ to 5 ‘strongly agree’. Items were formulated either positively (items 1, 3, 5) or negatively (items 2, 4, 6). The total score ranged from 6 to 30; a higher total score indicated the self-referred ability to produce a positive adaptation response when facing adverse situations.

1. Smith, Bruce W., Dalen et al. The brief resilience scale: assessing the ability to bounce back. International journal of behavioral medicine. 2008;15(3):194-200. doi: 10.1080/10705500802222972

Coping style

The cognitive, emotional, and behavioral way for dealing with problems were assessed using the Coping Scale [7], a self-report questionnaire consisting of 13 items. Each item was rated on a 4-point Likert Scale ranging from 1 ‘not true about me’ to 4 ‘mostly true about me’. The total score ranged from 13 to 52; a higher total score indicated the use of adaptive coping strategies.

1. Hamby S, Grych J, Banyard VL. Life paths measurement packet. 2013. Sewanee, TN: Life Paths Research Program.

Due to the lack of Italian versions of the BRS and Coping Scale, two researchers translated them into Italian independently; the two versions were compared to obtain a single draft. The draft of the BRS and Coping scale was presented to a native English speaker who translated the Italian versions back into English. The new English version was compared with the original English version, to highlight possible divergences and to obtain the final Italian version of both scales.
