# Supplementary material for: Subjective cognitive failures and their psychological correlates in a large Italian sample during quarantine/self-isolation for COVID-19
Source: Neurol Sci. 2021 Apr 29;42(7):2625–35. doi: 10.1007/s10072-021-05268-1 (PMC8082482; doi:10.1007/s10072-021-05268-1)
Supplement: Supplementary file 2 — (DOCX 17 kb). [file 10072_2021_5268_MOESM2_ESM.docx]

Supplemental Material 2. Mean, Standard Deviation, Cronbach’s alpha and Factors from principal components analysis (PCA) of Perceived Memory and Attentional Failures Questionnaire (PerMAFaQ), Brief Resilience Scale (BRS) and Coping Scale.

1. Mean, Standard Deviation, Cronbach’s alpha

|  | Mean (Standard deviation) | Cronbach’s alpha |
| --- | --- | --- |
| PerMAFaQ | 17.1 (6.5) | 0.883 |
| BRS | 21.1 (4.7) | 0.878 |
| Coping Scale | 33.4 (5.1) | 0.738 |

1. Factors of the Perceived Memory and Attentional Failures Questionnaire (PerMAFaQ) provided from Principal Components Analysis (PCA)

The PCA for PerMAFaQ revealed a two-factor model: the first factor (F1) explained 52.3% of variance, and included items related to attentive failures (items: 2,3,4,5,6). The second factor (F2) explained 12.5% of variance, and included items representing memory failures (items: 1,7,8,9)

|  | F1 (attentive failures) | F2 (memory failures) |
| --- | --- | --- |
| Item 6 | .821 | .262 |
| Item 4 | .785 | .014 |
| Item 3 | .783 | .320 |
| Item 2 | .704 | .394 |
| Item 5 | .694 | .418 |
| Item 8 | .154 | .844 |
| Item 9 | .140 | .760 |
| Item 7 | .349 | .726 |
| Item 1 | .422 | .545 |

1. Factors of the Coping Scale provided from Principal Components analysis

The PCA for Coping Scale revealed 4 eigenvalues exceeding 1, accounting for 61.6% of variance. The first factor (F1) explained 26.3% of variance, and included items related to capacity of seeing the positive side of the situation, seeing the humor in it (items: 2,5,8,10). The second factor (F2; related to the capacity of thinking about the problem from a different point of view, spending time trying to understand what happened), the third factor (F3, related to changing habits and lifestyle) and fourth factor (F4; related to the capacity of making compromises and waiting problem out) explained 13.8%, 12.2%, 9.3% of variance

|  | Factor 1 | Factor 2 | Factor 3 | Factor 4 |
| --- | --- | --- | --- | --- |
| Item 5 | .824 | .182 | -.011 | -.061 |
| Item 10 | .758 | -.091 | .180 | .216 |
| Item 2 | .633 | .335 | -.032 | -.130 |
| Item 8 | .576 | -.014 | .341 | .317 |
| Item 3 | .333 | .714 | -.022 | .022 |
| Item 4 | .432 | .674 | .101 | .012 |
| Item 1 | -.295 | .646 | .110 | .225 |
| Item 12 | .098 | .544 | .371 | -.223 |
| Item 9 | .220 | -.202 | .732 | .110 |
| Item 6 | .028 | .338 | .682 | .221 |
| Item 13 | -.035 | .424 | .682 | -.193 |
| Item 11 | .087 | .229 | -.085 | .778 |
| Item 7 | .023 | -.158 | .178 | .738 |
