# Supplementary material for: Subjective cognitive failures and their psychological correlates in a large Italian sample during quarantine/self-isolation for COVID-19
Source: Neurol Sci. 2021 Apr 29;42(7):2625–35. doi: 10.1007/s10072-021-05268-1 (PMC8082482; doi:10.1007/s10072-021-05268-1)
Supplement: Supplementary file 3 — (DOCX 16 kb). [file 10072_2021_5268_MOESM3_ESM.docx]

Supplemental Material 3.

1. Percentage of Healthcare workers and Non- Healthcare workers responding to complain cognitive failures from sometimes to always during quarantine/self-isolation

| PerMAFaQ | Healthcare workers | Non-medical workers |
| --- | --- | --- |
|  |  |  |
| Item 1 | 27.4 | 29.2 |
| Item 2 | 15.7 | 17.2 |
| Item 3 | 20.3 | 20.5 |
| Item 4 | 26.2 | 24.2 |
| Item 5 | 18.3 | 21.1 |
| Item 6 | 30.9 | 33.4 |
| Item 7 | 31.4 | 32.8 |
| Item 8 | 33.5 | 38.3 |
| Item 9 | 29.6 | 33.1 |
| Average | 25.9 | 27.7 |

PerMAFaQ, Perceived Memory and Attentional Failures Questionnaire

1. Percentage of people who work at office, people who work by smart-working and people who do not work during quarantine/self-isolation responding to complain cognitive failures from sometimes to always during quarantine/self-isolation

| PerMAFaQ | People who do not work | People who work by Smart-working | People who work at office |
| --- | --- | --- | --- |
|  |  |  |  |
| Item 1 | 29.1 | 27.5 | 32 |
| Item 2 | 17.7 | 15.8 | 16 |
| Item 3 | 20.5 | 21.5 | 17.3 |
| Item 4 | 23.9 | 24.6 | 26.7 |
| Item 5 | 20.7 | 20.6 | 20.5 |
| Item 6 | 34.7 | 32.3 | 27.3 |
| Item 7 | 33 | 32 | 31.8 |
| Item 8 | 39.6 | 34.7 | 35.9 |
| Item 9 | 34.4 | 29.7 | 31.8 |
| Average | 28.2 | 26.5 | 26..5 |

PerMAFaQ, Perceived Memory and Attentional Failures Questionnaire
